# Attention-enhanced dilated convolution for Parkinson’s disease detection using transcranial sonography

**DOI:** 10.1186/s12938-024-01265-5

**Published:** 2024-07-31

**Authors:** Shuang Chen, Yuting Shi, Linlin Wan, Jing Liu, Yongyan Wan, Hong Jiang, Rong Qiu

**Affiliations:** 1 School of Computer Science and Engineering, Central South University, No.932 South Lushan Road, Changsha, 410083 Hunan China; 2 Department of Neurology, Xiangya Hospital, Central South University, Changsha, 410083 China; 3 Key Laboratory of Hunan Province in Neurodegenerative Disorders, Central South University, Changsha, 410083 China; 4 Hunan International Scientific and Technological Cooperation Base of Neurodegenerative and Neurogenetic Diseases, Changsha, 410083 China; 5 National Clinical Research Center for Geriatric Disorders, Xiangya Hospital,, Central South University, Changsha, 410083 China; 6 National International Collaborative Research Center for Medical Metabolomics, Central South University, Changsha, 410083 China

**Keywords:** Parkinson’s disease, Transcranial sonography, Deep learning, Computer-aided diagnosis, Attention mechanisms, Movement disorders

## Abstract

**Background:**

Transcranial sonography (TCS) plays a crucial role in diagnosing Parkinson's disease. However, the intricate nature of TCS pathological features, the lack of consistent diagnostic criteria, and the dependence on physicians' expertise can hinder accurate diagnosis. Current TCS-based diagnostic methods, which rely on machine learning, often involve complex feature engineering and may struggle to capture deep image features. While deep learning offers advantages in image processing, it has not been tailored to address specific TCS and movement disorder considerations. Consequently, there is a scarcity of research on deep learning algorithms for TCS-based PD diagnosis.

**Methods:**

This study introduces a deep learning residual network model, augmented with attention mechanisms and multi-scale feature extraction, termed AMSNet, to assist in accurate diagnosis. Initially, a multi-scale feature extraction module is implemented to robustly handle the irregular morphological features and significant area information present in TCS images. This module effectively mitigates the effects of artifacts and noise. When combined with a convolutional attention module, it enhances the model's ability to learn features of lesion areas. Subsequently, a residual network architecture, integrated with channel attention, is utilized to capture hierarchical and detailed textures within the images, further enhancing the model's feature representation capabilities.

**Results:**

The study compiled TCS images and personal data from 1109 participants. Experiments conducted on this dataset demonstrated that AMSNet achieved remarkable classification accuracy (92.79%), precision (95.42%), and specificity (93.1%). It surpassed the performance of previously employed machine learning algorithms in this domain, as well as current general-purpose deep learning models.

**Conclusion:**

The AMSNet proposed in this study deviates from traditional machine learning approaches that necessitate intricate feature engineering. It is capable of automatically extracting and learning deep pathological features, and has the capacity to comprehend and articulate complex data. This underscores the substantial potential of deep learning methods in the application of TCS images for the diagnosis of movement disorders.

**Graphical Abstract:**

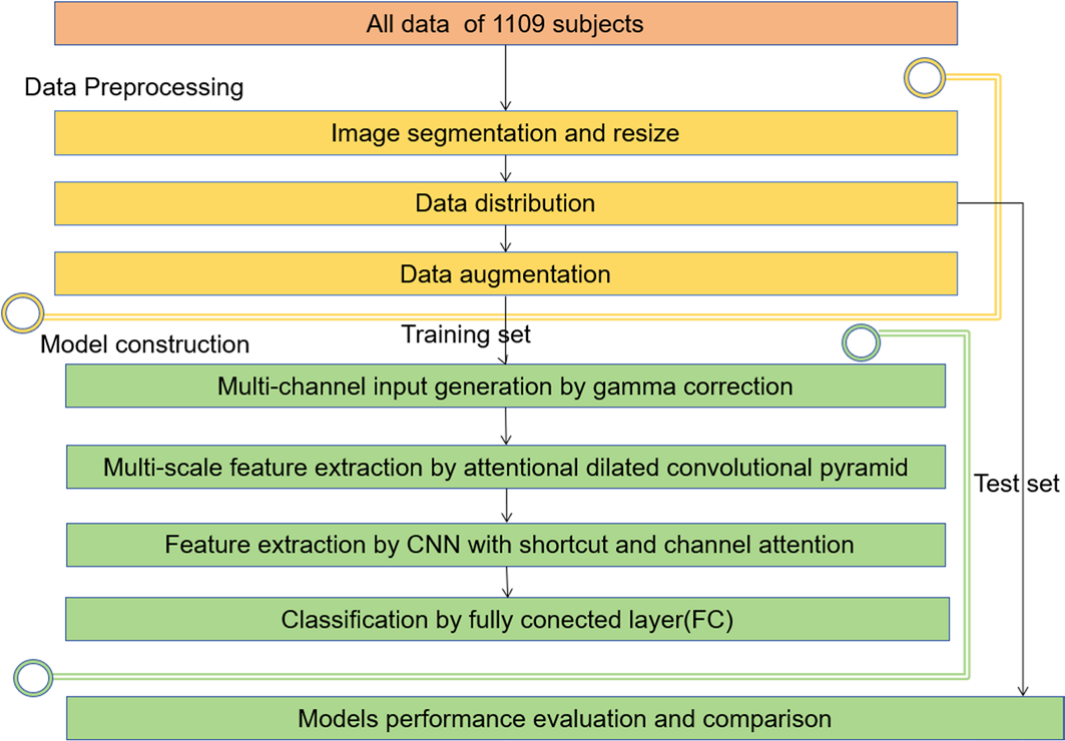

## Introduction

Parkinson’s disease (PD) is a common neurodegenerative disease that currently affects over six million individuals [1]. With the improvement in medical diagnosis and treatment levels and the progressive population ageing, the number of patients with PD is increasing, displaying an epidemic trend [2, 3]. The reduction in the labor force imposes a significant load on families and the society, severely affects the patients’ social function and quality of life. Additionally, PD’s clinical manifestations vary substantially, with numerous motor and non-motor symptoms. The clinical diagnosis of PD remains challenging [4]. Accurate diagnosis is crucial for effective PD treatment.

Transcranial sonography (TCS) is real-time, cost-effective, and non-invasive, widely used in clinical practice [5, 6]. TCS provides new information on brain morphology, aiding in diagnosing various movement disorders. Evaluation variations in the brainstem and subcortical structures offers a basis for diagnosing and differentiating movement disorders. Compared with other imaging methods, TCS equipment is relatively inexpensive, convenient to operate, and non-invasive. Since the first demonstration of the effectiveness of TCS for diagnosing PD, many scholars have focused on diagnosing movement disorders using TCS. In 1995, Becker first described the relationship between substantia nigra (SN) hyperechogenicity and PD [7]. Studies have shown that TCS can distinguish PD from essential tremors [8], atypical parkinsonism syndrome [9, 10]. Recently, Wang et al. [11] indicated that lens-shaped hyperechoic regions may help distinguish PD from essential tremors, multiple system atrophy, and progressive supranuclear palsy. Despite its wide application value, relevant computer-aided methods have not been extensively studied.

Pauly et al. [12] were the first to explore 3D imaging technology in TCS to assist in diagnosing PD, pioneering an automatic 3D SN hyperechogenicity detection method based on random forests. Subsequently, Plate et al. [13] proposed a TCS-based Parkinson's disease diagnosis method using support vector machines, focusing on the side with a large volume of SN hyperechogenicity. Despite high sensitivity and specificity in distinguishing PD from healthy subjects, these methods faced challenges in clinical applications due to their reliance on specialized equipment, resulting in small datasets and immature research methods. Sakalauskas et al. [14] introduced a semi-automatic segmentation method for the midbrain region in TCS images, combining statistical shape models with intensity amplitude invariant edge detectors. The team further explored the application of TCS in early PD, proposing an image analysis system incorporating a segmentation algorithm and a decision support subsystem [15]. Fei et al. [16] evaluated different regions of interest in feature extraction for TCS-assisted PD. Thirusangu et al. [17] proposed a deep convolutional neural network based on the U-Net architecture for automated SN, combining a weighted binary cross-entropy loss function for semantic segmentation in TCS images.

In TCS diagnostic classification tasks, various manual feature extraction methods based on machine learning have been extensively explored. Chen et al. [18] presented a local image analysis method using a support vector machine classifier to extract local features from detected spots and watershed regions of half the midbrain. Gong and Shi [19] proposed a deep neural mapping large margin distribution machine algorithm for PD diagnosis, utilizing a deep neural network for kernel mapping and a joint training strategy. Xue et al. [20] introduced a single-modal cKRVFL + (cascaded Kernel-based Random Vector Functional Link network plus) algorithm based on TCS images, which is an improved RVFL + algorithm (Random Vector Functional Link network plus). Shen et al. [21] proposed a PD diagnosis method using a deep polynomial network, employing a network pruning strategy to address overfitting. Shi et al. [22] integrated multimodal data from TCS images and transcranial Doppler ultrasound, proposing a computer-aided diagnosis method based on multi-kernel learning. Ding et al. [23] established the foundation for applying deep learning methods in diagnosing PD using TCS images, focusing on evaluating the performance of the ResNet and DenseNet models.

While 3D TCS image-based research methods can address issues related to poor sound transmission, their development is limited due to immature imaging technology, reliability concerns, and stringent data requirements. Traditional manual feature extraction methods have achieved successes in TCS image analysis, but rely on complex processes and extensive preliminary annotation work, limiting their practical applications. Manual annotation of regions of interest depends on doctors' expertise, increasing subjectivity and uncertainty. Machine learning methods lack the ability to automatically extract deep features, making noise handling challenging and resulting in insufficient diagnostic performance. In contrast, deep learning methods can automatically learn and extract features from raw images, avoiding cumbersome manual processes [24]. They also possess stronger generalization capabilities, facilitating data expansion and being less affected by different ultrasound machine models [25]. Therefore, exploring deep learning methods in TCS image classification and diagnostic tasks is crucial, promising reliable and efficient technical support for early detection and precise treatment of PD.

This paper proposes an Attention-Integrated Multi-Scale Residual Network (AMSNet) model combining an attention mechanism and multi-scale feature extraction structure for PD diagnosis. The model utilizes both original TCS images and gamma-corrected images as multimodal inputs, incorporating convolutional attention for fusion and dimensionality reduction. By contrasting and fusing information across different scales, the model reduces noise impact, enabling precise capture of brightness information related to the diagnostic target and enhancing lesion area feature extraction. The residual network structure embedded with lightweight channel attention captures hierarchical and detailed texture features in TCS images while reducing parameters count and avoiding gradient issues. AMSNet employs a multi-scale feature extraction module for robust lesion area processing.

This study utilized the second-largest dataset in the field of TCS PD diagnosis. Although not the largest, the data’s considerable scale ensured sufficient sample size for training and validating deep learning models. Leveraging this dataset, this study comprehensively extracted key information from TCS images through deep learning methods, providing new ideas for PD diagnosis.

The contributions of this study are as follows:

I. It proposed an Attention-Integrated Multi-Scale Residual Network (AMSNet) model for PD detection in TCS images. AMSNet, tailored to the unique characteristics of TCS images, enhances the accuracy of assisted diagnostic algorithms for PD utilizing TCS images.
II. It combined the dilated convolution pyramid and channel-spatial attention mechanism to establish an attention-dilated convolution pyramid module to extract multi-scale information in the image and expand the receptive field.
III. It used second-largest image database currently in in the field of TCS Parkinson's disease diagnosis to make the experimental results more reliable methods.

## Results

### Dataset

This study investigated 1109 subjects who visited the neurology clinic and ward of Xiangya Hospital of Central South University between December 2020 and October 2023. They comprised 675 patients with PD and 434 healthy controls. All the subjects were from mainland China, conscious, and cooperative. They provided informed consent before participating in the TCS examination. The TCS images used in the database, including those in the normal (healthy controls) and abnormal categories (PD), were randomly divided into training, validation, and test sets in the ratio 6:2:2. All the images were resized to 224 × 224 pixel using bilinear interpolation. For the training set, use random horizontal flips, random rotations, random blurring, and random scaling operations to achieve fivefold data augmentation. The settings of the dataset are listed in Table 1*.*

**Table 1:** Data setting of TCS for training, validation, and test sets

Image type	Training	Validation	Test
Abnormal	2025	135	135
Normal	1300	87	87
Sum	3325	222	222

### Experimental setting

This paper implements the proposed AMSNet using the PyTorch framework. The relevant software and hardware experimental environment are listed in Table 2. During network training, a batch size of 4 is used, along with the AdamW optimizer. The initial learning rate is set to 1 × 10^−4^, and an adaptive algorithm based on training loss dynamically adjusts the learning rate. Weighted cross-entropy loss, which has been proven to have a good performance in the classification algorithm [26], was used to optimize the model parameters during the training process. Each model was trained for at least 50 epochs. After the loss had no obvious decrease, we stopped training, and the best model, with the highest accuracy on the validation dataset, was saved.

**Table 2:** Software and hardware experimental environment

Setting	Item	Configuration
Hardware	CPU	Xeon(R) silver 4214R
	GPU	RTX 3080 Ti
	GPU memory	24 GB
Software	Programming language	Python 3.9.7
	DL architecture	Pytorch 1.10.1

### Results and analysis

First, experiments were conducted on TCS image data to evaluate the effectiveness of the AMSNet method in detecting PD. Then, ablation experiments were performed on the three important modules applied in the model (MVGGC, ADCP, and SE block) to demonstrate its effectiveness. Finally, the results of this method were compared with those of previous studies. The performance indicators used for the evaluation were the sensitivity (Se.), specificity (Sp.), precision (Pre.), F-score (F1), and overall accuracy (Acc.) of the experimental results.

#### Model evaluation

**Fig. 1:**
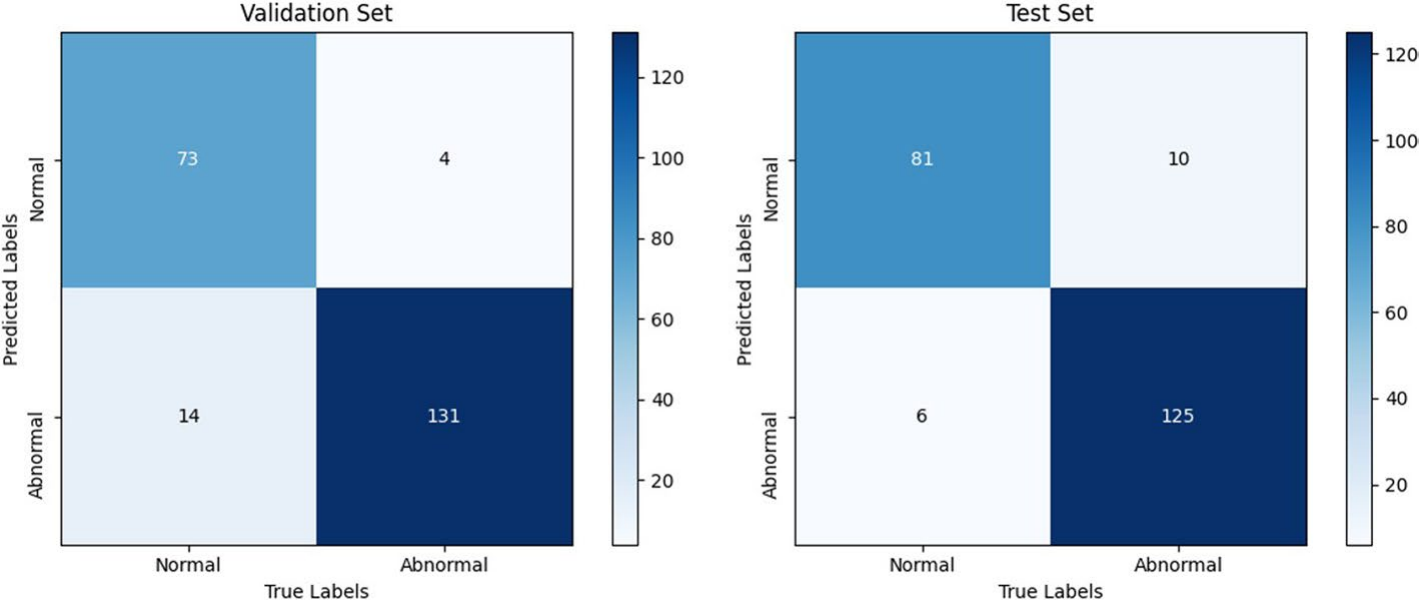
Confusion matrix for the result of AMSNet on validation and test sets

The AMSNet model was evaluated on the validation and test sets. The confusion matrix is shown in Fig. 1. For the validation set, 4 abnormal category images and 14 normal category images were misclassified. For the test set, 10 abnormal category images and 6 normal category images were misclassified. The evaluation index results of the experiments are listed in Table 3.

**Table 3:** Performance analysis of AMSNet on validation set and test set [In%]

Dataset	Category	Pre	Se	Sp	F1	Acc.
Validation set	Normal	94.81	83.91	97.04	89.02	91.89
	Abnormal	90.34	97.04	83.91	93.57	
Test set	Normal	89.01	93.1	92.59	91.01	92.79
	Abnormal	95.42	92.59	93.1	93.98	

The AMSNet model achieved Pre., Se., Sp. and F1 of 94.81% , 83.91%, 97.04%, and 89.02%, respectively, in the normal category of the validation set, and 90.34%, 97.04%, 83.91%, and 93.57%, respectively, in the abnormal category of the validation set. The overall accuracy was 91.89%. The AMSNet model achieved Pre., Se., Sp. and F1 of 89.01%, 93.1%, 92.59%, and 91.01%, respectively, in the normal category of the test set, and 95.42%, 92.59%, 93.1%, and 93.98%, respectively, in the abnormal category of the test set. The overall accuracy was 92.79%. The difference in Acc. between the validation and test sets did not exceed 1%. This indicated that the model exhibited good robustness.

#### Ablation experiments

We verified the effectiveness of the MVGGC layer, SE block, and ADCP layer in the AMSNet model. The results of the ablation experiments are listed in Table 4. In these experiments, we examined the performance of the model in the abnormal class, which was the PD class. In the first three experiments, the MVGGC layer, SE block, and ADCP layer were added to the backbone. In the fourth experiment, the ADCP layer was replaced with max pooling. In the fifth experiment, the SE block was replaced with a basic block in Resnet. In the sixth experiment, the MVGGC layer in AMSNet was deleted.

**Table 4:** Results of the ablation experiments on the three modules of AMSNet [In%]

Modules			Pre	Se	Sp	F1	Acc
MVGGC	SE	ADCP					
			90.37	90.37	85.06	90.37	88.29
√			92.97	88.15	89.66	90.49	88.74
	√		93.75	88.89	90.8	91.25	89.64
		√	94.49	88.89	91.95	91.95	90.09
√	√		91.91	92.59	87.36	92.25	90.54
√		√	95.28	89.63	93.1	92.37	90.99
	√	√	94.66	91.85	91.95	93.23	91.89
√	√	√	95.42	92.59	93.1	93.98	**92.79**

**Fig. 2:**
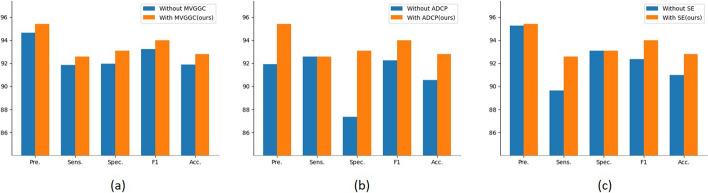
Performance analysis of ablation experiment on test set. **a** Displays the performance analysis for determining whether to add the MVGGC layer in AMSNet. **b** Displays the performance analysis for determining whether to use the ADCP layer in AMSNet. **c** Displays the performance analysis for determining whether to use SE in AMSNet

In ablation experiments, the model was trained on the training set and validation set, and tested on the test set. In Table 4, the first row shows the evaluation metrics of the basic Resnet model with an Acc. of 88.29%. When the MVGGC layer was added to the base model, Acc. increased to 88.74%. Similar results were obtained by adding the SE block and ADCP layer to the base model. The combined application of the SE block and ADCP layer yielded the largest improvement in the model, with Acc. increasing by 3.6%. Figure 2 mainly shows the result analysis of removing the MVGGC layer, SE block, and ADCP layer from AMSNet. Removing the ADCP layer from AMSNet resulted in a 2.25% decrease in the Acc. of the model. To a certain extent, it was demonstrated that the extraction and combination of multi-scale features are of high significance for PD detection in TCS. The failure of the MVGGC layer and SE block also impacted the model. The impact of the MVGGC layer was less than those of the other two modules. This is likely to be a result of the fact that the MVGGC layer should be combined with the SE block to obtain better results. AMSNet combines the three modules and achieves good results.

#### Comparison with other deep learning models

To verify the performance of the AMSNet model in the TCS-based Parkinson’s diagnosis task, we compared seven basic deep learning models: MaxViT [27], RepViT [28], BotNet [29], CrossViT [30], Swin-T [31], Inception-V3 [32], DenseNet [33] and MedViT [34]. The comparative experimental results on the test set for the AMSNet framework and other deep learning models are summarized in Table 5.

**Table 5:** Performance analysis of the proposed AMSNet in conjunction with the other deep learning models on the test set [In%]

Model/framework	Pre	Se	Sp	F1	Acc
MaxViT	90.00	*93.33*	83.91	91.64	89.64
RepViT	82.89	*93.33*	70.11	87.8	84.23
BOTNet	88.98	83.70	83.91	86.26	83.78
CrossViT	90.23	88.89	85.06	89.55	87.38
SwinT	*93.63*	87.41	*90.8*	90.42	88.74
Inception_V3	88.15	88.15	81.61	88.15	85.59
DenseNet	91.54	88.15	87.36	89.81	87.84
MedViT	89.66	**96.3**	82.76	*92.86*	*90.99*
AMSNet	**95.42**	92.59	**93.1**	**93.98**	**92.79**

The italicized results are the results of the comparison experiment that performed better

Among the conventional deep learning models, MedViT exhibited superior performance, achieving the highest accuracy of 90.99% and notable Se., and F1 scores of 96.3% and 92.86%, respectively. Several models stood out in different evaluation metrics. SwinT topped in Pre. And Sp. with 93.63% and 90.8% and MaxViT, RepViT and MedViT performed better than our model in Sens. However, our proposed AMSNet model surpassed the performance of these conventional models in multiple metrics. The AMSNet model recorded remarkable Pre., Sp. and F1 scores of 95.42%, 93.1%, and 93.98%, respectively, surpassing the corresponding scores of the conventional deep learning models.

These experimental results clearly demonstrate that, in the TCS diagnosis PD task, the AMSNet model proposed in this study exhibits significantly superior performance compared to conventional deep learning models. The AMSNet model's balanced performance across various metrics underscores its effectiveness and robustness in addressing the TCS-based Parkinson's diagnosis task.

Based on the information provided in Table 6, CrossViT exhibits the shortest runtime of 578.29 ms, while RepViT has the lowest parameter count of 2.167 M, albeit with relatively inferior performance. Although MedViT achieves good performance, its parameter count and runtime are relatively high. In contrast, AMSNet achieves high accuracy while balancing computational costs, making it highly practical for real-world applications.

**Table 6:** Comparison of runtime and parameter quantity among different methods

Model/framework	Running time	Parameter quantity
MaxViT	2128.04 ms	24.445 M
RepViT	594.25 ms	2.167 M
BOTNet	479.42 ms	18.802 M
CrossViT	578.29 ms	6.650 M
SwinT	1494.10 ms	27.498 M
Inception_V3	975.44 ms	41.146 M
DenseNet	829.97 ms	6.956 M
MedViT	1358.65 ms	31.138 M
AMSNet	1000.46 ms	22.273 M

#### Comparison with previous studies

The performance of AMSNet was compared with that of previous PD studies using TCS images. It is important to note that owing to the differences in datasets, methods, and validation techniques, the comparison of the results was biased. The results of the comparison are presented in Table 7. Most previous studies were based on 73-D feature extraction information from images to classify TCS. Moreover, the amount of data was small. For the AMSNet method, with the support of a large amount of data, deep learning can be used directly to extract multi-scale depth features from TCS images. As evident from Table 7, the AMSNet method surpassed the other methods in achieving an outstanding overall accuracy score. This underscores the significance and efficacy of extracting multi-type deep features, and the targeted improvements made in this paper with regard to image and pathological features are both effective and indispensable.

**Table 7:** Comparison of the proposed AMSNet with the models in the previous studies

	Dataset	Input	Acc. (%)
Shen et al. [21]	76 PD patients and 77 normal controls	73-D feature vector (for more details about statistical features	86.95
Xiaoyan et al.[16]	76 PD patients and 77 normal controls	73-D feature vector (for more details about statistical features	76.43
Shi et al. [22]	15 PD patients and 18 normal controls	73-D feature vector (for more details about statistical features	84.85
Shi et al. [20]	76 PD patients and 77 normal controls	73-D feature vector (for more details about statistical features	81.74
Ding et al. [23]	854 PD patients and 775 normal controls	Image	88.04
Ours	675 PD patients and 434 normal controls	Image	92.79

## Conclusion

This paper introduces the AMSNet method, a novel approach for diagnosing PD using TCS images, aiming to assist clinicians in making more precise diagnostic decisions. The key advantages of the AMSNet method lie in its utilization of deep learning techniques, rendering the model highly generalizable and scalable. Furthermore, the method effectively extracts multi-scale deep features from TCS images and employs an attention mechanism to modulate complex feature maps. The AMSNet method surpasses previous machine learning algorithms and current general-purpose deep learning models in diagnosing PD using TCS images. Ablation studies demonstrate the efficacy of the three introduced modules. Compared to previous methods, our approach exhibits superiority in terms of overall accuracy scores, offering new perspectives for future medical image-based diagnostic methods.

## Discussion

PD, a prevalent movement disorder, necessitates precise diagnosis for effective treatment and rehabilitation. TCS diagnostic information has demonstrated unique value in diagnosing Parkinson’s disease. However, due to the complexity of pathological features in TCS, inconsistent criteria for pathological manifestations, and the traditional analysis methods' heavy reliance on doctors' prior knowledge and operational experience, it is particularly urgent to provide objective and accurate decision support for the diagnosis of Parkinson's disease in TCS. Existing Parkinson's disease diagnostic aids based on TCS images predominantly rely on machine learning techniques. These methods often require tedious manual annotation by doctors and manual extraction of statistical and textural features. This approach not only relies on complex feature engineering, but also often fails to effectively capture deep features in images, resulting in insufficient diagnostic accuracy and limited automation and generalization performance. In contrast, deep learning possesses the ability to automatically extract deep image features without complex feature engineering, thus exhibiting significant advantages in the field of image processing. However, existing universal deep learning methods do not consider the specific image characteristics in TCS and the pathological manifestations of movement disorders. Currently, there is insufficient research on the application of deep learning algorithms in the field of Parkinson's disease diagnosis based on TCS images.

In light of this, the present study explores the application of deep learning methods in the analysis of TCS images. By analyzing the key points and challenges in diagnosing Parkinson's disease using TCS images, we propose the Attention-integrated Multi-Scale Network (AMSNet), a residual network model combining attention mechanisms and multi-scale feature extraction. AMSNet incorporates both the original TCS images and gamma-corrected images as multimodal inputs, enabling a better capture of brightness information relevant to the diagnostic target. To address the challenge of irregular feature regions in TCS and the importance of area information, AMSNet introduces a multi-scale feature extraction module that robustly handles the morphological features of lesion areas. Furthermore, the integration of channel-spatial attention for fusion and dimensionality reduction, along with contrastive fusion across different scales, can mitigate the impact of noise in ultrasound images while focusing more on lesion areas. AMSNet employs a residual network structure with lightweight channel attention. The residual structure allows the model to stack deep networks to fully capture hierarchical and detailed textures in TCS images, while avoiding gradient vanishing or explosion issues. Meanwhile, the lightweight channel attention module optimizes these features while preventing overfitting due to excessive parameters. Experimental validation demonstrates that AMSNet exhibits superior performance in diagnosing Parkinson's disease using TCS images, outperforming traditional methods. Each module demonstrates its necessity, providing doctors with a more automatic and accurate diagnostic aid and providing strong technical support for precise treatment of Parkinson's disease.

In practical applications, AMSNet can assist doctors in initial screening and diagnosis by rapidly analyzing key information in patients’ TCS images and providing diagnostic suggestions. This improves diagnostic accuracy and efficiency, reduces doctors' workload, and enables timely treatment. Additionally, AMSNet can be used during PD treatment to monitor image changes, in assessing treatment effects, and adjust plans accordingly. AMSNet algorithm offers valuable references for other TCS-related research, potentially aiding in diagnosing other neurological diseases.

Although AMSNet demonstrates exceptional performance in diagnosing Parkinson’s disease using TCS images, it still faces some limitations:

1. Data dependency: The model’s performance depends on the quality and quantity of training data. The current dataset’s small size may limit generalization, especially for new or special cases.
2. Interpretability: Deep learning models often lack intuitive explanations, affecting doctors' trust in diagnosis.
3. Computational resources: AMSNet requires high computational resources for training and inference, limiting its application in resource-constrained medical institutions.
4. Technological updates: Continuous development in deep learning means AMSNet may be replaced by more advanced models. Maintaining technological updates is crucial for sustainable development.

Future research could explore more data sources and enhancement methods to improve AMSNet’s generalization. Introducing interpretability techniques could improve doctors' trust. Optimizing the model structure and reducing computational resource demands could make AMSNet more suitable for various medical institutions. Staying updated with emerging technologies and integrating novel findings into AMSNet’s refinement is essential.

## Method

### Overall architecture

**Fig. 3:**
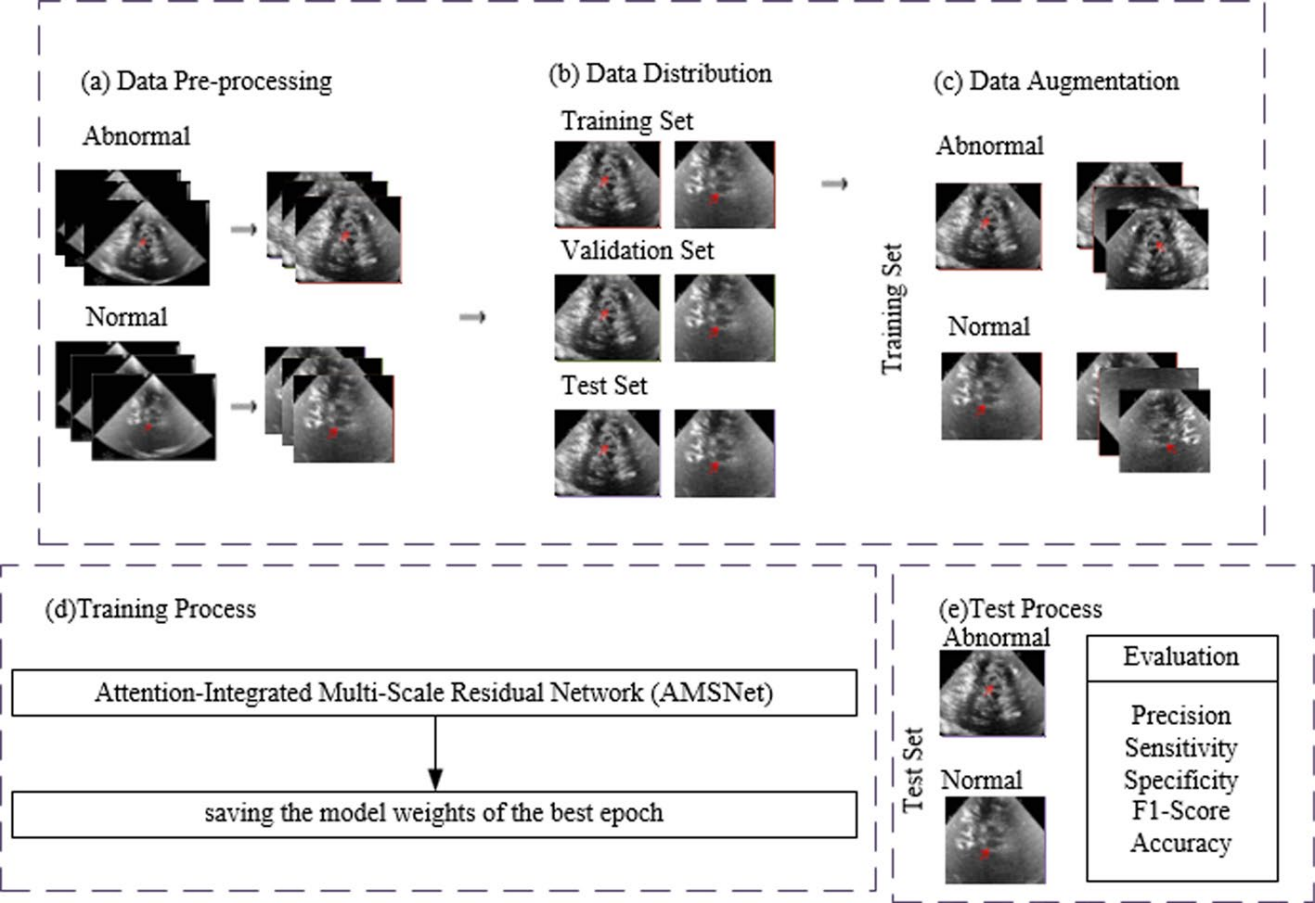
The entire process of the AMSNet framework. **a** displays the image preprocessing stage. The dataset is then divided in **b**. In **c**, the training set data are expanded. In **d**, the weight of the epoch with the best performance in the validation set during the training phase is saved. In **e**, the performance of the model is evaluated in the test set

The entire process of AMSNet framework is illustrated in Fig. 3.

**Fig. 4:**
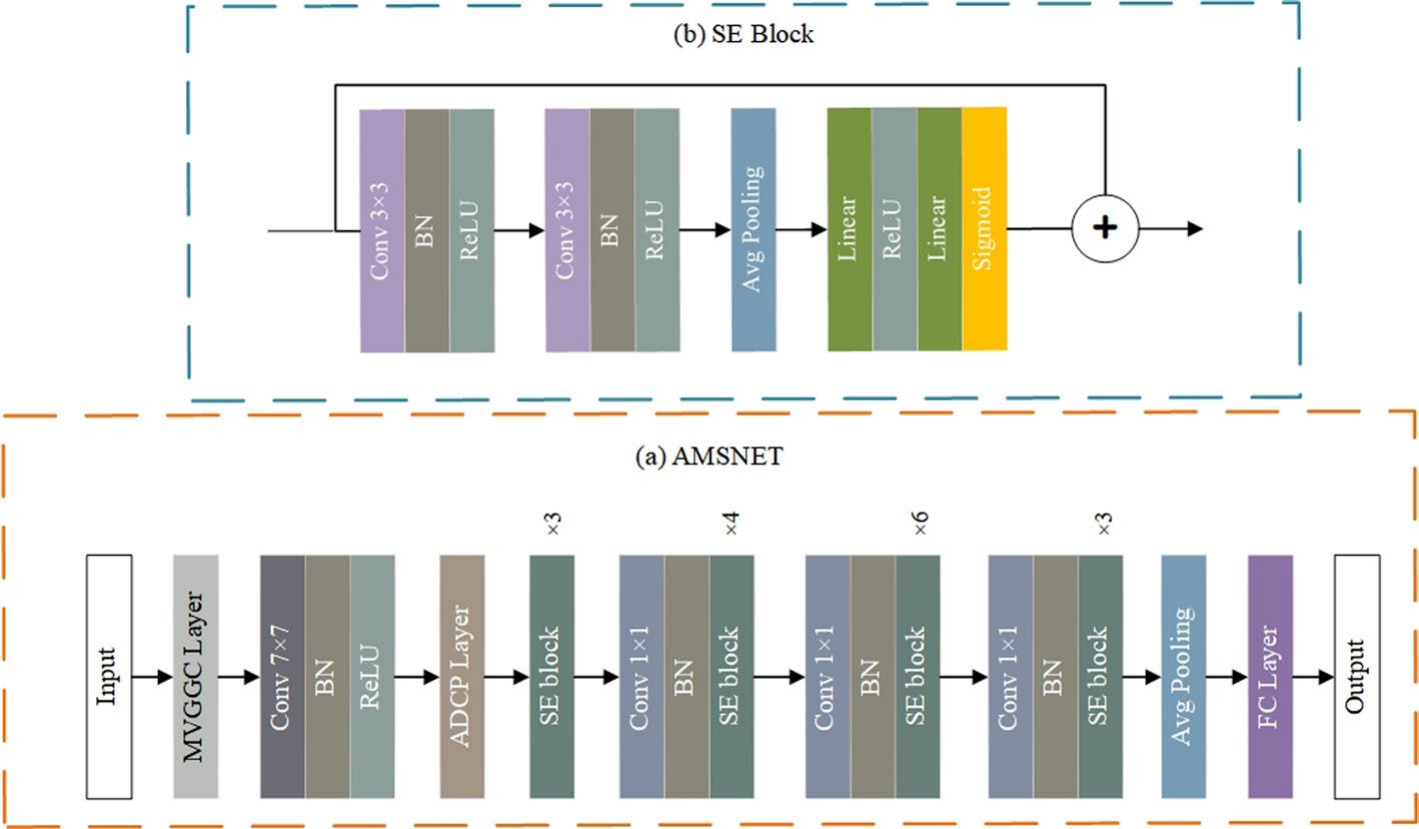
The structure of the proposed AMSNet framework. **a** Displays the simplified structure of AMSNet. **b** Describes the structure of the squeeze-and-excitation (SE) block in detail

Step 1 In Fig. 3a, the ultrasound parameter annotation, skull, and other noise information in the image are segmented and removed through preprocessing.
Step 2 In Fig. 3b, all the images are divided into training set, validation set, and test set.
Step 3 In Fig. 3c, the images of the training set are augmented.
Step 4 In Fig. 3d, AMSNet is trained on the established dataset, and the weights of the model in the epoch with the best performance in the validation set are stored.
Step 5 In Fig. 3e, the model parameters with the best performance in the validation set are used to test the test set images, and the final classification results are obtained. The network architecture of AMSNet, depicted in Fig. 4, is designed with the purpose of thoroughly capturing intricate hierarchical information and delicate texture details in TCS images for precise assisted diagnosis of PD. The model incorporates a residual network structure, which enables the stacking of deeper layers, thereby facilitating a deeper exploration of crucial image information. Through residual connections, the model effectively mitigates the issues of gradient vanishing or exploding, thereby enhancing stability and accuracy during the training process.

To address the pervasive issue of noise interference in TCS data, AMSNet specifically incorporates a Multi-View Generation layer via Gamma Correction (MVGGC). This layer generates images with varying brightness characteristics by applying gamma correction to the original TCS images and subsequently concatenates them into a multi-channel input for the model. This approach not only enhances the model's ability to capture fine-grained details, but also effectively mitigates the impact of noise, thereby improving the model's robustness. Furthermore, considering the multi-scale nature of TCS data, AMSNet incorporates an Attentional Dilated Convolutional Pyramid (ADCP) module. This module constructs feature maps at multiple scales and utilizes a channel-spatial attention mechanism to dynamically adjust the weights of these feature maps. This enables the extraction and fusion of multi-scale features, allowing the model to comprehensively understand the morphological and structural characteristics of the lesion area. This, in turn, provides more accurate and comprehensive information for subsequent diagnostic analysis. To further enhance the model's feature representation capabilities, AMSNet employs a Squeeze-and-Excitation (SE) module. This module learns the interdependencies between feature maps and adaptively recalibrates the weights of individual channels. This allows the model to emphasize features that are crucial for the diagnosis of PD, thereby facilitating the identification of key information in complex TCS data. Consequently, the accuracy and reliability of diagnosis are improved.

This section comprises the following parts: the multi-view generation module is described in ″Multi-View Generation by Gamma Correction (MVGGC)″ section. The establishment of the attention-dilated convolution pyramid module is detailed in ″Attentional dilated convolutional pyramid (ADCP)″ section. The multi-channel attention mechanism is explained in ″Channel attention module″ section.

### Multi-view generation by gamma correction (MVGGC)

In general, the positivity rate of the hyperechoic region in the substantia nigra, the rate of lenticular hyperechogenicity of the nucleus, and the width of the third ventricle are deemed crucial features in diagnosing movement disorders. In particular, for PD diagnosis, the shape information, area size, and distribution of the hyperechoic region in the substantia nigra are essential for accurately interpreting TCS images. However, due to the varying quality of images, especially in low-quality ultrasound images, the identification of pathological features is often limited. Therefore, enhancing image contrast and clarity, thus rendering the boundaries between hyperechoic regions and background information more distinct, is of significant importance for improving diagnostic accuracy.

Gamma correction, an effective image enhancement technique, is widely used to adjust the brightness and contrast of images. By precisely tuning the gamma value, it enables precise control over different tone ranges in an image, thereby enhancing its visual effect and making the differences between dark and bright areas more prominent. Additionally, gamma correction ensures that darker regions of the image do not become completely black, preserving image details and providing more comprehensive and accurate data for subsequent feature extraction and analysis.

**Fig. 5:**
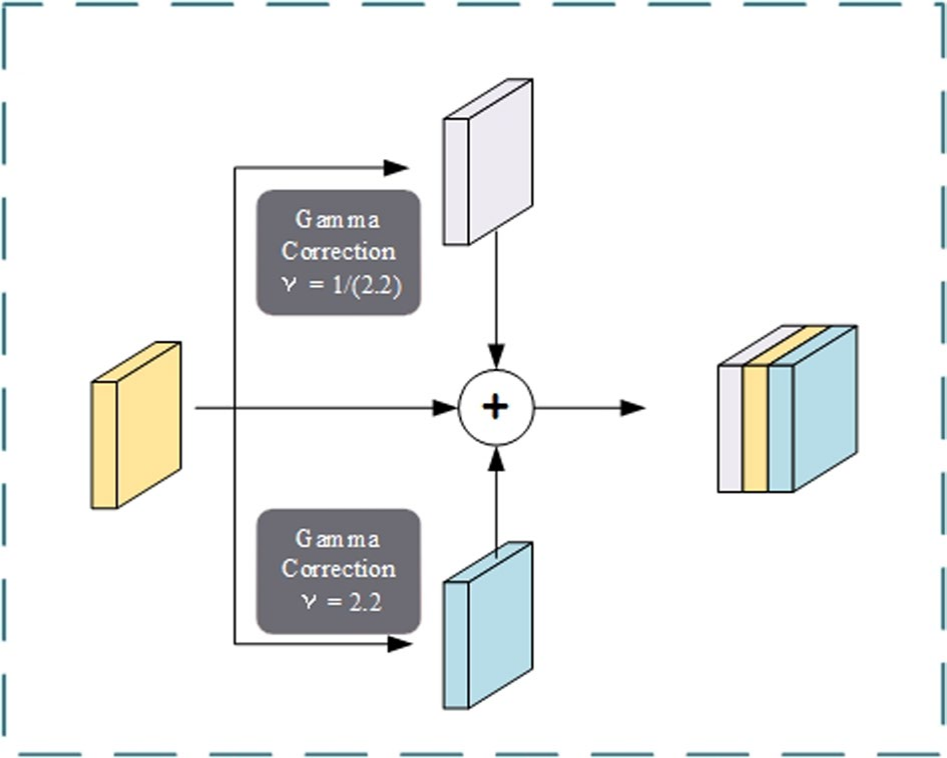
The structure of the MVGGC layer. In this layer, the original image is gamma corrected to generate multi-view information

Therefore, this paper introduces a Multi-View Generation by Gamma Correction (MVGGC) module [35]. This module generates multiple images with varying brightness characteristics using gamma correction and concatenates them into a multi-channel input, as depicted in Fig. 5. This approach not only enriches the input information for the model, but also helps improve the overall image quality and information retention. Consequently, by extracting and analyzing features from these multi-view images, the model's diagnostic capabilities for movement disorders such as PD can be further enhanced. The formula for gamma correction is as follows:

1 $$\hat{X}_{i} \, = \,f_{{{\text{GA}}}} \left( {X,\gamma } \right)\, = \,C \cdot \,\left( {\frac{X + \varepsilon }{C}} \right)^{\gamma } ,$$

where $X$ is the image to be adjusted, ${\widehat{X}}_{i}$ is the image generated by the gamma correction, $\gamma$ is the encoded or decoded gamma value, $\varepsilon$ is a constant multiplier, and $C$ is a constant matrix.

Different gamma correction coefficients have different effects on the image. According to the characteristics of the gamma curve, when the gamma correction coefficient is less than 1, the brightness of the filtered ultrasound image is higher than that of the original image, the dynamic range of the dark part of the image is extended, and the dynamic range of the bright part is compressed. When the gamma correction coefficient is higher than 1, the brightness of the filtered ultrasound image is reduced compared with that of the original image, the dynamic range of the dark part of the ultrasound image is compressed, and the dynamic range of the bright part is extended. Therefore, performing two gamma corrections on the original image can yield different information from the image and form an input of multiple views that can represent more abundant information in the ultrasound image. For each TCS image, the following three images (as shown in Fig. 5) are used: (1) the cropped original TCS image, (2) the gamma correction value of the TCS image is $1/2.2$, and (3) the gamma value is $2.2$. The output of the MVGGC layer is expressed as follows:

2 $$\hat{X}\, = \,\left( {\mathop {{\text{cat}}}\limits_{i = 1}^{n} f_{{{\text{GA}}}} \left( {X,\gamma_{i} } \right)} \right),$$

where cat represents the contact operation, ${f}_{\rm GA}$ is the gamma correction function and $\gamma$ is the encoded or decoded gamma value.

To obtain more feature information, a multi-view generation layer was added to the model. This method combines three gamma-corrected TCS images and incorporates the features of the TCS image. Compared with the method that uses only one view as the input, the MVGGC layer integrates different gamma-corrected images. It retains the features of the original TCS images while extracting additional potential features from each view.

### Attentional dilated convolutional pyramid (ADCP)

TCS data possess inherent multi-scale characteristics, which refer to the varying sizes, shapes, and levels of detail exhibited by pathological regions, such as hyperechoic regions in the substantia nigra, in medical images during TCS examination. These features differ with changes in the observation or analysis scale. Accurate identification and characterization of pathological regions require the ability of auxiliary diagnostic analysis models to capture and understand these subtle differences across multiple scales, posing a significant challenge in TCS image analysis. In deep learning, the ability to handle such multi-scale features is particularly crucial [36].

Atrous Spatial Pyramid Pooling (ASPP) [37], as a method of increasing the receptive field, effectively addresses the contradiction between receptive field expansion and resolution loss during image feature extraction. It enables the model to maintain a high resolution while acquiring broader contextual information, thus comprehending image content more comprehensively. The atrous spatial pyramid pooling structure further extends the application of atrous convolution by combining atrous convolution operations at different scales, effectively capturing multi-scale information in images [38–41]. This structure overcomes the limitations of single-scale feature extraction.

**Fig. 6:**
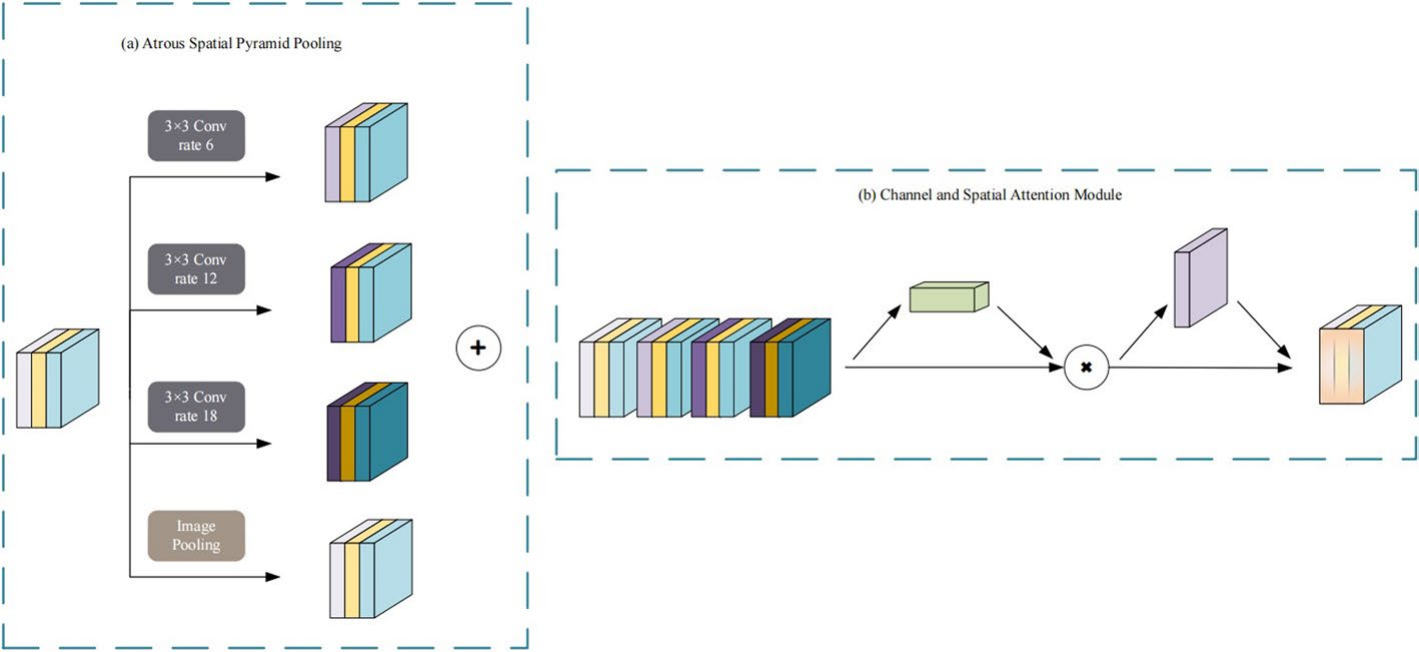
The structure of the ADCP layer. **a** Uses the dilated convolution pyramid to process multi-view information. **b** Combines the output in **a** with the attention mechanism

To better accommodate the complex demands of TCS image diagnostic analysis, this paper combines the atrous spatial pyramid pooling (ASPP) structure with the convolutional block attention module (CBAM) [42] and proposes the attention-dilated convolutional pyramid module (ADCP). The structure of the ADCP is illustrated in Fig. 6. This module employs a dual mechanism to capture multi-scale features and enhance the weights of regions of interest. On one hand, leveraging the characteristics of ASPP, ADCP is capable of capturing and fusing multi-scale features in images, obtaining richer and more comprehensive pathological information. On the other hand, with the aid of CBAM, the module can adjust the weights of feature maps precisely. By combining channel attention and spatial attention mechanisms, it generates channel attention feature maps and spatial attention feature maps, thereby achieving precise localization and information enhancement of regions of interest. Through the construction of the attention-dilated convolutional pyramid module, AMSNet can simultaneously extract multi-scale pathological features and enhance the weights of regions of interest, resulting in more accurate feature extraction from TCS images.

(1) Dilated spatial pyramid structure

We created convolutional layers with rates of [6, 12, 18]. Additionally, we added a branch that directly pools without a dilated convolution to construct a pyramid structure to expand the receptive field for features at different scales. The structure of the module is illustrated in Fig. 6a. Multiscale feature extraction is performed for $\widehat{X}$ to generate MSF. The computational process is expressed as follows:

3 $$\begin{array}{c}{\rm MSF}={\text{cat}}\left(\underset{i=1}{\stackrel{n}{\text{cat}}}\left({f}_{\text{DC}}\left(\widehat{X},{r}_{i}\right)\right),{f}_{\text{p}}\left(\widehat{X}\right)\right),\end{array}$$

where $\text{cat}$ denotes the contact operation, ${\text{f}}_{\text{DC}}$ is the dilated convolution operation, $\text{r}$ represents the expansion rate, and ${\text{f}}_{\text{p}}$ is the pooling operation.

(2) Channel spatial joint attention

To calculate the channel attention features more efficiently, it is necessary to compress the spatial dimension of the feature map and use maximum pooling and average pooling comprehensively. ${\text{I}}_{\text{avg}}^{\text{c}}$ and ${\text{I}}_{\text{max}}^{\text{c}}$ represent the average and maximum pooling features, respectively. The generated features are fed into a shared multi-layer perceptron (MLP) to produce a final channel attention feature map. To reduce the parameter overhead, the output of the hidden layer is ${W}_{1}\in {R}^\frac{c}{r}$. Here, $r$ is the scaling rate. Finally, the channel attention weight is outputted using the element-by-element summation method. The calculation process of the channel attention feature map is shown in Eq. (4):

4 $$\begin{aligned} {{\text{Att}}_{{\text{c}}} ({\text{I}})} & = \sigma \left( {{\text{MLP}}\left( {{{f}}_{{{\text{Ap}}}} \left( {{I}} \right)} \right) + {\text{MLP}}\left( {{{f}}_{{{\text{Mp}}}} \left( I \right)} \right)} \right) \\ {} & = \sigma \left( {{{W}}_{1} \left( {{{W}}_{0} \left( {{{I}}_{{{\text{avg}}}}^{c} } \right)} \right) + {{W}}_{1} \left( {{{W}}_{0} \left( {{{I}}_{{{\text{max}}}}^{c} } \right)} \right)} \right) \\ \end{aligned}$$

where $I$ is the input feature, ${f}_{\rm Ap}$ and ${f}_{\rm Mp}$ refer to average and max pooling, respectively, MLP stands for multi-layer perceptron, $\sigma$ is a sigmoid function,${W}_{1}$, ${W}_{2}$ represent the two layers of weights in the MLP.

(3) Spatial attention

We use the spatial relationships between features to generate spatial attention maps to complement channel attention, which pays more attention to which location in the data is more effective. In the spatial attention module, average and maximum pooling are performed in the channel dimension. ${I}_{\rm avg}^{c}$ and ${I}_{\text{max}}^{c}$ represent the average and maximum pooling features, respectively. The resulting features are concatenated using convolution operations to produce a spatial attention feature map. Finally, the feature map output is obtained by the spatial attention module through a sigmoid function, which can be expressed as Eq. (5):

5 $$\begin{aligned} {\text{Att}}_{\text{s}}(\text{I})& =\sigma \left({C}_{7\times 7 }\text{Cat}\left({{f}}_{\text{Ap}}\left({I}\right);{{f}}_{\text{Mp}}\left({I}\right)\right)\right)\\ & \left.=\sigma \left({C}_{7\times 7}\left({I}_{\text{avg}}^{s};{I}_{\text{max}}^{s}\right.\right)\right),\end{aligned}$$

where $\text{I}$ is the input feature, $\text{cat}$ represents the contact operation, ${\text{f}}_{\text{Ap}}$ and ${\text{f}}_{\text{Mp}}$ refer to average and max pooling, respectively, $\upsigma$ is a sigmoid function, ${\text{C}}_{7\times 7}$ is a convolution operation with a convolution kernel size of 7.

(4) The convolutional block attention module

CBAM is a module that combines channel attention and spatial attention. Its structure is shown in Fig. 6b. The CBAM module adjusts the attention weights of the multi-scale feature map to generate an enhanced feature map. The specific calculation formulas are shown in Eq. (6) and Eq. (7):

6 $${\text{MSF}}^{\prime } \, = \,{\text{Att}}_{c} \left( {{\text{MSF}}} \right)\, \cdot \,{\text{MSF}},$$

7 $${\text{MSF}}^{\prime \prime} = {\text{Att}}_{s} \left( {{\text{MSF}}^\prime} \right) \cdot {\text{MSF,}}^\prime$$

where MSF′ is the multi-scale feature map, MSF″ is the attention multi-scale feature map after adjusting the weights, Att_c_ and Att_s_ are the channel attention map and spatial attention map, respectively, and $\cdot$ represents element-by-element multiplication.

The ADCP layer proposed in this study aims to optimize the feature extraction process. The ADCP layer first uses ASPP to extract multi-scale features from the feature map, which helps the model capture pathological details at different scales. Subsequently, the CBAM module combines these features through channel and spatial joint attention mechanisms, allowing the model to adaptively focus on important features. Finally, dimensionality reduction and fusion are performed through a convolutional layer, which not only reduces the computational complexity but also retains key information. This design can more effectively capture multi-scale information in images, thereby improving the accuracy of pathological feature extraction. Although this method does not directly enhance the resolution or pixel accuracy of the image, it significantly improves the quality of the processing results in the early stages of feature processing, providing a more reliable auxiliary tool for medical image analysis. At the same time, it also provides a solution with reference value for other medical impact and image processing research with the same application scenarios.

### Channel attention module

ResNet is a series of CNN models. This network structure hinders the conveyance of global information regarding the input data to the end of the model. The loss of this part of global information affects the performance of the model. In AMSNet, the SE block with a good channel weight distribution is selected to strengthen the importance between the channel features. The structure of the SE block is shown in Fig. 4b. The SE block comprises two operations: squeezing and excitation. The squeeze operation encodes the entire spatial feature into a global feature using global average pooling to generate the channel statistics. An excitation operation is used to obtain the channel importance of two fully connected layers, a dimensionality reduction layer, and an increasing layer. The final channel weights are obtained by the sigmoid activation function. An increase in the number of parameters of the SE block results in a minimal increase in the amount of computation, using low time and computational consumption to assign weights to the importance of the channel information for each feature map. Because convolution operates only in a local space, it is difficult to obtain sufficient information to extract the relationship between channels. Moreover, its impact on the previous layers in the network is more severe. An SE block was used to extract the channel weights to improve the features. The squeeze operation encodes the entire spatial feature of a channel into a global feature. It is implemented using global average pooling. Global average pooling can be defined as:

8 $${\text{SQ}}_{{{k}}} = \frac{1}{{{{H}} \times {{W}}}}\sum\nolimits_{i = 1}^{{{H}}} {\sum\nolimits_{j = 1}^{{{W}}} {{{F}}_{{{k}}} \left( {i,j} \right)} } ,$$

where ${k}\in \text{1,2},3$ represents different channels, ${F}$ is the input data, and $\text{SQ}$ is generated by global average pooling.

The SE module in the excitation phase processes these global eigenvalues through a bottleneck structure consisting of two fully connected layers. First, the first fully connected layer reduces the number of channels to reduce computational complexity and the number of parameters. Subsequently, the introduction of the ReLU activation function provides the model with nonlinear characteristics, enabling it to learn complex interactions between channels. Next, the second fully connected layer restores the number of channels to the original dimension. The weights are normalized using the sigmoid activation function to reflect the importance of different channels for the final feature representation. The calculation process of the excitation phase is shown in Eq. (9):

9 $$\begin{array}{c}\text{EX}=\delta \left({{W}}_{2}\cdot \text{ReLU} \left({{W}}_{1}\cdot \text{SQ}\right)\right),\end{array}$$

where $\sigma$ is a sigmoid function, ${{W}}_{1}$, and ${{W}}_{2}$ are the weights of the two fully connected layers.

Finally, the learned activation value weight of each channel was multiplied by the original feature to complete the recalibration of the original feature in the channel dimension.

In the basic block structure of Resnet, this study adopts a method combined with the SE module. After the two convolutional layers of the Resnet basic block, the feature map is recalibrated through the SE module, and finally the processed feature map is added to the input of the residual connection to obtain the final output result. The operation of the final output result $\text{RSE}$ can be expressed as:

10 $$\begin{array}{c}\text{RSE}=\widetilde{\text{F}}\left({n}\right)+F\left({n}-1\right),\end{array}$$

where $\text{F}\left({n}-1\right)$ is the output of the ${\left({n}-1\right)}_{{th}}$ layer and $\widetilde{\text{F}}\left({n}\right)$ is the output of the SE module of the ${\text{n}}_{{th}}$ layer.

The method of combining ResNet and SE block was adopted in our study. This helped the network understand and weigh the characteristic responses of each channel better. By combining these two architectures, dual advantages can be obtained in feature extraction: retaining features through the deep structure of ResNet and improving the quality of features through the attention mechanism of the SE block. This combination achieves significant performance improvements in image-processing tasks. It is particularly effective for processing complex visual data.

## Data Availability

The data analyzed in this study are subject to the following licenses/restrictions: datasets analyzed in this study are not publicly available. Further information about the datasets is available to researchers upon reasonable request to the author (HJ). Requests to access these datasets should be directed to HJ, jianghong73868@126.com.
